# From the Editor’s Desk

**Published:** 2011-04

**Authors:** Andries Brink

**Affiliations:** The Cardiocascular Journal of Africa

The *Cardiocascular Journal of Africa* (CVJA) is making great strides but faces considerable challenges. It is now well accredited in all of the major databases in the world and is widely read. The increase in readership over the past year was 33% and 3 000 articles are downloaded monthly via Pubmed LinkOut. This reflects usage of only four years of the CVJA, which has a dataset of 400 full-text articles in Pubmed.

However, we are facing a bottleneck due to insufficient appropriate reviewers for the submitted articles, and this is causing a delay in publishing these articles. It appears that South African reviewers take greater pride in reviewing articles for foreign journals. The CVJA is as well rated as any foreign journal. We are now as a matter of policy also registering authors as reviewers.

Please note that because of the backlog in printed publications we have provided authors with the opportunity to publish ahead of print. This implies that your article will appear in an electronic version such as PubMed and elsewhere. There will, however, be an additional charge of R1 000 for African authors and R3 000 for overseas authors.

We now have CrossCheck available to check for any suspected plagiarism in articles.

Ek wil graag outeurs nooi om ook wetenskaplike werk voor te lê vir publikasie in CVJA in Afrikaans of ander inheemse Afrika tale. Dit sal gelyke aandag geniet as die engelse artikels met dieselfde soort erkenning en verspreiding. Die tydskrif wil baie graag jonger navorsers op die gebied van hartsiektes aanmoedig om navorsingswerk op te skryf. Ons oorweeg tans ’n toekenning vir ’n artikel wat in Afrikaans of ’n inheemse taal geskryf is en toepaslik is vir Afrika siektes en omstandighede.

Indien daar geen eweknie beoordeelaars (peer reviewers) beskikbaar is vir ’n bepaalde artikel, word die manuskrip terug gestuur aan die outeur.

If no reviewers are available for reviewing the article, it will unfortunately have to be sent back to the author.

We thank the following South Africans who reviewed articles for the CVJA during 2010:

Julia Aalbers, M Abelson, J Badenhorst, Piet Becker, Megan Bester, Paul Brink, Geoffrey Candy, R Chauke, Ashley Chin, Daneel Dietrich, Stefan du Plessis, Anna-Mart Engelbrecht, Rajiv Erasmus, Rafique Essop, M Faadiel Essop, Mieke Faber, Julia Goedecke, Vladimir Grigorov, Dave Harris, Mbuilu Jody, James Ker (jun), James Ker (sen), John Lawrenson, Melanie Louw, Leoné Malan, Maurice Mars, J Marx, Indres Moodley, M Mpe, Cephas Musabayane, M Ntsekhe, Andrzej Okreglicki, Brian Rayner, Paul Rheeder, Saartjie Roux, Robert Schall, Aletta Schutte, R Scott-Millar, Y Seedat, Brandon Shaw, Karen Sliwa, Jan Smedema, Cornelius Smuts, Harris Steinman, Krisela Steyn, Nicky Sulzer, H Theron, Nico van der Merwe, Lynette van der Merwe, Willie van Heerden, Jacques van Rooyen, W Venter, Corinna Walsh, Paul Williams, Angela Woodiwiss, Liesl Zäuhlke, Makhosazane Zungu

